# Facilitators of and Barriers to Resilience Among Black Children and Youth in Canada and the United States: Protocol for a Scoping Review

**DOI:** 10.2196/80859

**Published:** 2025-10-20

**Authors:** Emmanuel Akwasi Marfo, Gervin Ane Apatinga, Bukola Salami

**Affiliations:** 1 Department of Community Health Sciences Cumming School of Medicine University of Calgary Calgary Canada

**Keywords:** Black children and youth, resilience, Canada, United States, well-being

## Abstract

**Background:**

Black children and youth disproportionately experience systemic discrimination, racism, stereotypes, and academic streaming, which negatively affect their well-being. Although they often demonstrate high aspirations and resilience, dominant narratives remain deficit-based, emphasizing challenges over strengths. The limited research on resilience among Black children and youth in Canada restricts the development of informed policy and future scholarship.

**Objective:**

This scoping review aims to identify, describe, and synthesize existing evidence on the facilitators of and barriers to resilience among Black children and youth in Canada and the United States. Given the shared histories of anti-Black racism and similarly structured systems, such as education, health care, and child welfare, the scope of this study extends to the United States to offer relevant insights that can help inform policy, practice, and future research in both contexts.

**Methods:**

This review will be guided by a methodological framework for scoping reviews proposed by Arksey and O’Malley and advanced by Levac and colleagues. We searched 4 multidisciplinary electronic databases (CINAHL, Ovid MEDLINE, ERIC, and PsycINFO) for relevant reports. The Thesis and ProQuest Dissertations and Theses Global database will also be searched for unpublished student theses and dissertations. Two independent researchers will complete the screening process. Using the PRISMA-ScR (Preferred Reporting Items for Systematic Reviews and Meta-Analyses Extension for Scoping Reviews) guidelines, the findings will be synthesized quantitatively and qualitatively through thematic analysis.

**Results:**

Data screening is ongoing and will be followed by data extraction, analysis, and the drafting of the final manuscript for peer-reviewed publication.

**Conclusions:**

This scoping review will generate critical insights into the multifaceted factors that support or hinder resilience among Black children and youth in Canada and the United States. By centering intersectionality and community-engaged approaches, the findings will inform equity-oriented policies, culturally responsive practices, and future research that amplifies the strengths of Black young people. Ultimately, this scoping review will contribute to disrupting systemic inequities and supporting the flourishing of Black children and youth across diverse contexts.

**International Registered Report Identifier (IRRID):**

DERR1-10.2196/80859

## Introduction

### Background

Black children and youth disproportionately encounter systemic discrimination, racism, stereotypes, and academic streaming [1-3], which are challenges that lead to disparities in education, health, and economic opportunity. These disparities include low educational attainment, overrepresentation in the criminal justice system and welfare system, and poor physical and mental health [4-6]. Collectively, these negative outcomes reinforce persistent socioeconomic disparities between Black youth and their peers from other racial and ethnic backgrounds.

Despite these challenges, research shows that Black children and youth often hold high aspirations for their future [7,8]. However, systemic discrimination and racism can hinder their ability to pursue and realize these goals. One study found that students belonging to visible racial or ethnic minority groups were more likely to aspire to attend a university compared to their peers belonging to nonvisible racial or ethnic minority groups [9], a finding that has been replicated in other studies [10]. Even as they strive to achieve their aspirations, Black children and youth are often subjected to deficit-based narratives that reinforce negative stereotypes [8,11].

In response to these barriers and challenges, many Black children and youth engage in strategies that foster resilience, which is the capacity to thrive amid adversity [12]. Resilience has been conceptualized in multiple ways, including the ability to access and effectively use psychological, social, cultural, and physical resources [13] as well as a set of interconnected processes between individuals and systems that enable the use of these resources to achieve positive outcomes [14]. These conceptualizations align with the biopsychosocial model of human development proposed by Bronfenbrenner [15]. While some of the definitions mentioned earlier emphasize individual capacities, others highlight the systemic and relational processes involved in fostering resilience. For this study, we define resilience in Black children and youth as (1) a dynamic process; (2) a set of capacities; and (3) a range of outcomes that enable them to adapt, cope, and flourish despite ongoing exposure to systemic adversity. This highlights the role of cultural identity, community support, and lived experience in shaping how resilience is expressed and sustained among Black children and youth.

Although there is a growing body of research focused on the experiences of Black children and youth in Canada and the United States [10,16-18], targeted studies on resilience remain relatively sparse. In the Canadian context, especially, there is a notable lack of synthesized evidence on the factors that support or hinder resilience. While recent reviews have explored mental health and general health among Black youth [19-21], these studies represent only a subset of the broader social, structural, and cultural conditions shaping resilience. On the basis of a preliminary review of the literature and to the best of our knowledge, no existing literature, no existing literature reviews have systematically examined the factors influencing resilience among Black youth in Canada. This represents a critical gap. Accordingly, this review aims to fill that gap by synthesizing evidence on the complex and intersecting dynamics that shape resilience in this population.

Due to the limited Canadian literature, we expand the scope of this review to include the United States, providing a broader base of evidence to inform policy, practice, and future research in both countries. While Canada and the United States differ in population size, health care systems, immigration pathways, and social policies [22-24], they share critical historical and structural features [25]. Both are settler-colonial states with enduring legacies of anti-Black racism and face parallel challenges in advancing racial equity in health, education, and social services. Their geographic proximity, common language, and history of cross-border policy exchange further support the value of reviewing literature from both contexts. In light of these contextual differences and similarities, there are opportunities to illuminate resilience processes that may be transferable or adaptable to the Canadian context.

### Objectives

By examining findings from Canada and the United States while attending to their distinct systemic and cultural environments, this review aims to inform Canadian policy through both context-specific and comparative insights.

Therefore, this review aims to identify, describe, and synthesize existing evidence on the facilitators of and barriers to resilience among Black children and youth in Canada and the United States. Importantly, we recognize the heterogeneity within Black populations, including the diverse experiences of first-, second-, and later-generation migrants from continental Africa and the Caribbean as well as descendants of survivors of the trans-Atlantic slave trade [26]. While their backgrounds are varied, shared racialization and phenotypic similarities often expose them to similar patterns of discrimination and exclusion.

## Methods

### Theoretical Lens

This scoping review will be guided by an intersectionality-based theoretical lens, which provides a critical framework for understanding how overlapping systems of oppression, such as race, class, gender, immigration status, and age, interact to shape the lived experiences of Black children and youth [27-29]. This lens enables a more nuanced exploration of resilience by recognizing that it is shaped not only by individual or cultural factors but also by the intersections of structural and systemic inequities. By applying this approach, we will be sensitive to the complexity and diversity within Black communities, moving beyond one dimensional analyses and highlighting how multiple identities and contexts contribute to both vulnerabilities and sources of strength [30,31].

### Researchers’ Positionality

Our research team includes Black Ghanaian, Nigerian, and Canadian scholars with disciplinary backgrounds in nursing and health geography, representing diversity across gender and generational age. The project is supervised by a Canada Research Chair in Black and Racialized People’s Health with recognized expertise in the well-being of Black children and youth. Two undergraduate Black and racialized youth research assistants are actively involved in the project, contributing lived experience and critical reflections that inform this study’s design and focus. We have established a Black youth advisory committee for stakeholder consultation and feedback on data interpretation and synthesis. Our intersecting positionalities as racialized, migrant, and health equity scholars enable us to critically engage with the structural conditions shaping the well-being of Black youth and children. We recognize the complexities of our proximity to and distance from this population and will engage in reflexive dialogue to address how our identities and assumptions shape the research process. We reject claims to absolute objectivism and instead embrace a standpoint rooted in care, equity, and justice.

### Design

This review will be guided by the methodological framework for scoping reviews proposed by Arksey and O’Malley [32] and advanced by Levac et al [33]. This framework involves five key stages: (1) formulating a research question; (2) identifying relevant studies; (3) selecting studies; (4) organizing and extracting data; and (5) compiling, summarizing, and reporting the findings.

#### Formulating the Research Question

The research question for this scoping review was developed in response to gaps identified in the existing literature on the well-being of Black children and youth. Specifically, we seek to answer the following research question: What individual, community, relational, and cultural factors facilitate or hinder resilience among Black children and youth in the context of systemic adversity in Canada and the United States?

#### Identifying Relevant Studies: Search Strategy

This scoping review will use a search strategy designed to locate both published and unpublished empirical reports. A preliminary search on relevant electronic databases (CINAHL, Anthropology Plus, Criminal Justice Abstracts, LGBTQ+ Source, MEDLINE, Health Source: Nursing/Academic Edition, and ERIC) was conducted using a search strategy on May 19, 2025 (Multimedia Appendix 1).

The research team consulted a health sciences librarian to refine the search strategy. The preliminary search revealed significant overlaps between domain-specific databases. To ensure practical feasibility and comprehensive content coverage, the initial list of databases was reduced to 4 major electronic databases. This decision was made to maintain methodological rigor while ensuring a manageable and transparent review process by prioritizing databases that offered the most comprehensive and nonredundant coverage.

Specifically, CINAHL and Ovid MEDLINE were chosen for their health focus (eg, medicine, clinical sciences, public health and epidemiology, nursing, biomedical sciences, health services research, psychology and behavioral sciences, and medical education), while ERIC and PsycINFO were selected for their focus on education and psychology (eg, educational psychology, gender, sexuality, cultural studies, stress and resilience, social work, and developmental psychology). This combination ensures a robust multidisciplinary approach, as these major databases already include a significant portion of the sociological, kinesiological, and social work literature relevant to the topic. For example, MEDLINE and PsycINFO cover not only their core disciplines but also content on social phenomena and behavioral sciences, which encompasses much of the research on resilience and systemic adversity.

To minimize publication bias, the team will also search for gray literature, including unpublished theses, dissertations, and relevant nongovernmental organizations and government documents. In addition, the reference lists of included studies will be reviewed to identify any further sources. The search strategy will be rerun before the final analysis and manuscript submission to capture any newly published relevant studies for inclusion in the final report (Multimedia Appendix 2).

#### Selecting Studies: Inclusion and Exclusion Criteria

We will include original primary research studies with quantitative, qualitative, or mixed method designs using the population, concept, and context framework (Table 1 and Table 2) [34,35]. We will include studies conducted in English from 2000 to the present to reflect the widespread adoption of multisystemic and intersectional resilience frameworks in the 21st century and ensure the relevance of generated evidence for contemporary policy and practice, particularly following heightened awareness and advocacy around racial injustices. Excluding French and other non-English studies may limit the comprehensiveness of this scoping review. While this decision reflects practical resource constraints, we acknowledge the transnational nature of Black communities and Canada’s bilingual context. Consequently, valuable perspectives, particularly from Francophone Canadians, may be missed. We will exclude nonresearch reports (eg, editorials, commentaries, and opinions). Conference abstracts will also be excluded from formal extraction, analysis, and synthesis, as inconsistencies between an abstract and the full report are common [36]. However, relevant abstracts may be examined informally to identify emerging topics, geographic trends, or shifts in research focus over time. This information may be used to inform the discussion of gaps and future research priorities.

**Table 1:** Population, concept, and context (PCC) framework.

PCC element	Definition
Population	Black children and youth aged between 0 and 24 years (broadly defined to include diverse populations from continental Africa, the Caribbean, and descendants of those enslaved through the trans-Atlantic slave trade)
Concept	Resilience, which includes processes, capacities, and outcomes among Black individuals despite exposure to significant stress or hardship (Table 2)
Context	Canada and the United States

**Table 2:** Operationalized dimensions of resilience for study screening.^a^

Resilience dimension	Operational definition	Indicators for inclusion	Example
Process	A dynamic process of adapting to, coping with, or resisting adversity over time	Describes how Black children and youth adapt to systemic adversity; longitudinal or developmental analysis of coping or adaptation; includes mechanisms or trajectories	A study examining how Black adolescents navigate school discrimination over several years
Capacities (resilience factors)	Individual, relational, or contextual characteristics that support or promote positive adaptation in the face of adversity	Mentions protective or promotive factors such as self-efficacy, cultural identity, community support, family cohesion, mentorship, and religious or spiritual beliefs	A study exploring the role of Black cultural identity in coping with racial profiling
Outcomes	Observable behavioral, psychological, developmental, or social manifestations of resilience	Includes positive outcomes such as academic engagement, leadership, self-esteem, positive identity, social competence, and adaptive functioning	A study reporting improved academic performance despite exposure to racism

^a^Studies that use the term *resilience* superficially or metaphorically without linking it to defined outcomes, capacities, or processes will be excluded.

#### Organizing and Extracting Data: Data Management, Evidence Extraction, and Reference Management

After completing the database search, we will remove all duplicates and transfer the reports to Covidence (Veritas Health Innovation) software for systematic reviews. We will conduct a 2-step screening process on the articles. First, 2 independent reviewers will screen the titles and abstracts to identify relevant reports using the inclusion and exclusion criteria. Second, the same reviewers will assess the full texts of potentially relevant studies. Throughout the screening process, the research team will meet to discuss conflicts and will resolve them through consensus. To ensure consistency and minimize subjectivity, particularly given the multidimensional nature of resilience, reviewers will use a full-text screening tool (Multimedia Appendix 3). This guide includes clear inclusion rules and examples of resilience-related processes and outcomes (eg, effective coping, cultural adaptation, social connectedness, and religious coping) based on the definition outlined in the population, concept, and context framework.

A data extraction tool will be piloted to determine its suitability and will be modified if necessary for use in data extraction (Multimedia Appendix 4). Following that, the independent reviewers will extract data from all reports that will be included in this review. The extracted data will include specific details about the author names and publication dates, country of origin, purpose and aims of the study, design and methods, type of adversity, findings, resilience facilitators, resilience barriers, implications, and research limitations [37]. If possible, we will contact the authors of the included reports via their email contact information if there are missing data [38]. Missing data will be categorized as “not reported” if attempts to retrieve them are not successful. In alignment with the option not to appraise the quality of included studies by Arksey and O’Malley [32], we will not conduct quality appraisal, as our goal is not to make judgments about the quality of evidence but to map the breadth of literature on this topic.

#### Compiling, Summarizing, and Presenting the Findings

We will present the results of the screening process and reasons for the exclusion of reports from the full-text screening in the final report using the PRISMA-ScR (Preferred Reporting Items for Systematic Reviews and Meta-Analyses Extension for Scoping Reviews) [39] guidelines. We will use descriptive statistics to analyze the frequencies of the characteristics of studies included in this review and the quantitative findings from quantitative and mixed methods studies. The frequencies of included studies with similar characteristics, such as country of origin, range of publication dates, and study designs, will be tallied and presented using graphs and tables.

Guided by an intersectionality-based theoretical lens [27], we will analyze the extracted data from qualitative studies using a thematic approach [40] in NVivo (Lumivero) software. Initially, the project lead and research assistants will conduct open inductive coding of the first 5 extracted reports to categorize emerging evidence on resilience facilitators and barriers. The broader research team will then meet to review and discuss the initial codes, refining them collaboratively into a preliminary coding framework.

This coding framework will be used to guide analysis of the remaining data while still allowing for inductive modification as new themes emerge. Codes will be grouped into themes and subthemes based on recurring patterns, contextual factors, and intersectional considerations. The team will engage in regular analytical meetings, ensuring that multiple perspectives inform the interpretation of findings and reduce individual bias. Reflexive discussions with the 5-member advisory youth committee will also be used to interrogate assumptions and positionality throughout the analytic process. To ensure the review maintains relevance for Canadian policy and practice, we will categorize and analyze Canadian and US studies separately during data charting. Thematic comparisons across both contexts will be conducted to identify shared patterns as well as context-specific distinctions. Data from the United States will be interpreted with attention to its unique contextual conditions while also highlighting elements that may be transferable or adaptable to the Canadian landscape.

We will use a narrative approach to organize findings by study design (qualitative, quantitative, or mixed methods). Themes will be organized into overarching categories such as individual, relational, community, and structural levels, in alignment with our biopsychosocial and intersectionality-informed lens. This layered synthesis will allow us to present an integrated understanding while noting where and how methodological differences shape insights. We will synthesize the findings into a final report with general conclusions and recommendations as well as limitations.

### Ethical Considerations

This study did not require ethics approval because data will be obtained from already published sources and does not include direct involvement of human participants. However, we recognize that ethical considerations remain important, especially given the sensitive nature of topics such as childhood trauma, racism, mental health, sexual orientation, disability, and other marginalized social identities. We are mindful of the potential for research to inadvertently reinforce stigma, a risk we seek to mitigate by emphasizing community strengths. Our approach aims to present the evidence with respect and sensitivity, centering the lived experiences of Black children and youth within their broader sociohistorical contexts.

### Reporting Standards

This protocol has been submitted for registration and will be embargoed on the Open Science Framework. This will become publicly accessible upon journal publication [41].

## Results

**Figure 1:**
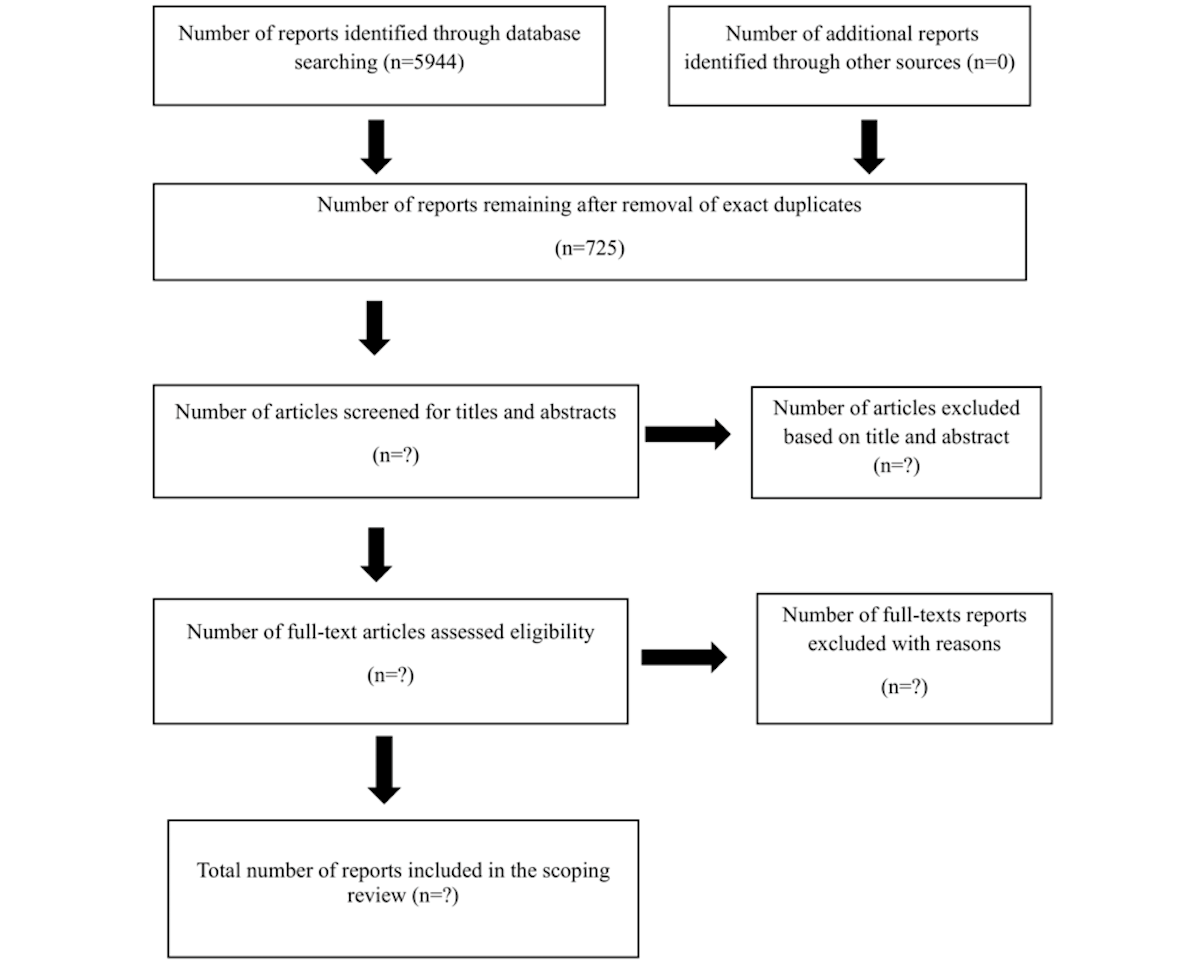
PRISMA-ScR (Preferred Reporting Items for Systematic Reviews and Meta-Analyses Extension for Scoping Reviews) flowchart.

The research team finalized the conceptualization of this scoping review protocol in June 2025. The comprehensive literature search was completed on July 16, 2025, in consultation with a health sciences librarian. This search yielded 5944 reports, of which 725 (12.20) duplicates were identified and removed using Covidence (Figure 1). We anticipate completing data screening, selection, extraction, and analysis by September 2025. The final report will be submitted for publication in a peer-reviewed, open-access journal.

## Discussion

### Anticipated Findings

This scoping review is expected to identify and synthesize key facilitators of and barriers to resilience among Black children and youth in Canada and the United States. We anticipate identifying a broad range of individual, relational, community, and structural factors that enable or constrain resilience. These factors include, but are not limited to, cultural identity, family cohesion, mentorship, experiences of racism, and systemic barriers in education and health care. We also expect that resilience processes and outcomes will vary based on intersecting social identities (eg, gender, immigrant status, and socioeconomic position), revealing the need for nuanced and tailored interventions.

Unlike previous reviews, which focused primarily on mental health and general health outcomes among Black children and youth [19-21], this review positions resilience as both a process and an outcome, incorporates an intersectionality lens, and situates findings within structural and cultural contexts, offering a more holistic understanding of resilience. By situating resilience within structural and cultural contexts, this work addresses critical gaps in the literature and builds on earlier efforts to understand how systemic inequities shape Black youth resilience. Finally, this review aligns with ongoing national efforts to improve outcomes for Black communities in Canada, particularly in response to enduring racial inequities and those highlighted during the COVID-19 pandemic [42].

### Strengths and Limitations

A key strength of this review is its comprehensive and multidisciplinary approach, informed by health, education, social science, and community perspectives. This study is also strengthened by its grounding in Black-led research, community engagement, and critical theoretical frameworks, including intersectionality and the biopsychosocial model developed by Bronfenbrenner. Our study has a few limitations. Excluding articles published in French may limit the comprehensiveness of this scoping review, given that Canada is a bilingual country. Consequently, the findings will primarily reflect evidence available in English-language studies. In addition, the concept of resilience is inherently complex and interpreted differently across various disciplines [43]. This inherent variability could pose challenges during the screening and data extraction phases when identifying resilience facilitators and barriers. To mitigate this, we will apply operationalized dimensions of resilience (Table 2) and use a reflexive, consensus-driven approach within the research team during data screening and extraction.

### Implications for Future Research

Future research should adopt bilingual or multilingual approaches to incorporate Francophone communities and perspectives, thereby enhancing inclusivity and representativeness. Expanding resilience research across diverse cultural and structural contexts will be critical for developing effective, equity-driven policies and programs.

### Dissemination Plan

To maximize the impact and accessibility of our findings, we will disseminate results through both academic and community channels. These include publication in a peer-reviewed journal, presentations at relevant conferences (eg, the Black Child and Youth Summer Institute), and cocreated knowledge translation products (eg, podcasts and summary briefs). Our Black youth advisory committee will play an active role in codeveloping these outputs to ensure they are accessible, culturally resonant, and action oriented for advocacy organizations, educators, community groups, and policymakers.

### Conclusions

By moving beyond deficit-oriented frameworks, this scoping review protocol centers the strengths, strategies, and structural contexts that shape how Black young people navigate systemic adversity. Guided by intersectionality and grounded in community-engaged scholarship, this review will offer a multidimensional synthesis of the processes, capacities, and outcomes that support or hinder resilience. In doing so, this study will inform culturally responsive, equity-driven policy and practice interventions that reflect the lived realities and aspirations of Black youth. The findings will contribute critical knowledge to disrupt systemic inequities and advance health, educational, and social justice for Black children and youth.

## Appendix

Multimedia Appendix 1 Preliminary search strategy.

Multimedia Appendix 2 Final comprehensive search strategy.

Multimedia Appendix 3 Full-text screening tool.

Multimedia Appendix 4 Data Extraction Table.

## Abbreviations

PRISMA-ScR: Preferred Reporting Items for Systematic Reviews and Meta-Analyses Extension for Scoping Reviews
