# Supplementary material for: Facilitators of and Barriers to Resilience Among Black Children and Youth in Canada and the United States: Protocol for a Scoping Review
Source: JMIR Res Protoc. 2025 Oct 20;14:e80859. doi: 10.2196/80859 (PMC12583943; doi:10.2196/80859)
Supplement: Multimedia Appendix 1 [file resprot_v14i1e80859_app1.docx]

**Multimedia Appendix 1: Preliminary Search Strategy**

**Electronic Databases**

A preliminary search on relevant electronic databases CINAHL, Anthropology Plus, Criminal Justice Abstracts, LGBTQ+ Source, MEDLINE, Health Source: Nursing/Academic Edition and Eric

**Population:**

“black or African American or African-American or black American"

"children or adolescents or youth or child or teenager"

**Concept**:

“resilience or resiliency or resilient or strengths or coping or hardiness or adaptation or grit or resistance or emotional stability"

**Context:**

"United States or America or USA or US or Canada"

**Search Strategy**

“black or African American or African-American or black American" AND "children or adolescents or youth or child or teenager" AND “resilience or resiliency or resilient or strengths or coping or hardiness or adaptation or grit or resistance or emotional stability" AND "United States or America or USA or US or Canada"
