# Supplementary material for: Facilitators of and Barriers to Resilience Among Black Children and Youth in Canada and the United States: Protocol for a Scoping Review
Source: JMIR Res Protoc. 2025 Oct 20;14:e80859. doi: 10.2196/80859 (PMC12583943; doi:10.2196/80859)
Supplement: Multimedia Appendix 2 [file resprot_v14i1e80859_app2.docx]

**Multimedia Appendix 2: Final Comprehensive Search Strategy**

**Databases:** CINAHL, OVID Medline, PsycINFO, ERIC

**Population:**

1. Black OR black OR African American OR African-American OR Black American OR Afro-American OR Afro American OR Afro-descendant OR Afrodescendant OR person of African descent OR people of African descent OR African ancestry OR African heritage OR African origin OR African diaspora OR Afro-Caribbean OR Afro Caribbean OR Caribbean Black OR Caribbean-American OR Caribbean American
2. child OR children OR adolescent OR adolescents OR youth OR youths OR teenager OR teenagers OR teen OR teens OR young people OR young person OR juvenile OR juveniles OR minors OR preteen OR preteens OR pre-adolescent OR pre-adolescents OR school-aged OR school age OR high school student OR middle school student OR young individual OR young population

**Concept:**

resilience OR resiliency OR resilient OR psychological resilience OR emotional resilience OR coping OR coping skills OR coping strategies OR stress management OR adaptation OR adaptive capacity OR adaptability OR hardiness OR psychological hardiness OR personal strengths OR strengths-based OR personal resources OR grit OR perseverance OR determination OR mental toughness OR resistance OR emotional stability OR emotional regulation OR emotion regulation OR self-regulation OR self-efficacy OR thriving OR bouncing back OR positive adaptation OR stress resistance

**Context:**

United States OR United States of America OR USA OR U.S.A. OR US OR U.S. OR America OR American OR Americans OR U.S. population OR U.S.-based OR United States-based OR North America OR Canada OR Canadian OR Canadians OR Canadian population OR North American OR Alabama OR Alaska OR Arizona OR Arkansas OR California OR Colorado OR Connecticut OR Delaware OR Florida OR Georgia OR Hawaii OR Idaho OR Illinois OR Indiana OR Iowa OR Kansas OR Kentucky OR Louisiana OR Maine OR Maryland OR Massachusetts OR Michigan OR Minnesota OR Mississippi OR Missouri OR Montana OR Nebraska OR Nevada OR New Hampshire OR New Jersey OR New Mexico OR New York OR North Carolina OR North Dakota OR Ohio OR Oklahoma OR Oregon OR Pennsylvania OR Rhode Island OR South Carolina OR South Dakota OR Tennessee OR Texas OR Utah OR Vermont OR Virginia OR Washington OR West Virginia OR Wisconsin OR Wyoming OR Ontario OR Quebec OR Nova Scotia OR New Brunswick OR Manitoba OR British Columbia OR Prince Edward Island OR Saskatchewan OR Alberta OR Newfoundland and Labrador OR Northwest Territories OR Yukon OR Nunavut
