# Supplementary material for: Facilitators of and Barriers to Resilience Among Black Children and Youth in Canada and the United States: Protocol for a Scoping Review
Source: JMIR Res Protoc. 2025 Oct 20;14:e80859. doi: 10.2196/80859 (PMC12583943; doi:10.2196/80859)
Supplement: Multimedia Appendix 3 [file resprot_v14i1e80859_app3.docx]

**Multimedia Appendix 3: Full-Text Screening Tool**

**Instructions:**

Include the article if “Yes” to all questions.

1. **Is the study a primary empirical research study (qualitative, quantitative, or mixed methods)?**

Yes_____________________________________________________________

No______________________________________________________________

Reviewer’s Notes____________________________________________________

1. **Is the population focused on Black children and youth (0–24 years)?***Relevant Notes: Include Black/African American/Caribbean children and youth even if not disaggregated by age subgroups (0–24)* Yes_____________________________________________________________

No______________________________________________________________

Reviewer’s Notes____________________________________________________

1. **Does the study focus on resilience (as factors and/or outcomes) in an adversity?**

*Relevant notes: Tick at least one of the categories below. If none are ticked, mark “No.”*

☐ Individual traits (e.g., self-esteem, self-efficacy, optimism)
☐ Social support (e.g., mentorship, peer/family support, measured or described in relation to resilience)
☐ Cultural identity (e.g., racial pride, connection to heritage)
☐ Behavioral outcomes (e.g., academic engagement, leadership)
☐ Emotional regulation (e.g., non-clinical coping, emotion management)
Yes_____________________________________________________________

No______________________________________________________________

Reviewer’s Notes____________________________________________________

1. **Was the study conducted in Canada and/or the United States?**

Relevant notes: *Include multi-country studies if they include data from Canada/US.*

Yes_____________________________________________________________

No______________________________________________________________

Reviewer’s Notes____________________________________________________

1. **Is the full-text available in English?**

Yes_____________________________________________________________

No______________________________________________________________

Reviewer’s Notes____________________________________________________

1. **Is the study published between the year 2000 and now?**

Yes_____________________________________________________________

No______________________________________________________________

Reviewer’s Notes____________________________________________________

1. **Is the article not a commentary, editorial, opinion piece, or review article?**

Yes_____________________________________________________________

No______________________________________________________________

Reviewer’s Notes____________________________________________________

**Important Notes (Studies that do not meet the criterion for resilience)**

- Exclude studies that focus exclusively on risk factors or mental illness (e.g., depression, trauma) without linking to resilience or adaptive outcomes.
- Exclude studies on economic outcomes (e.g., household income, employment status) unless they are presented as a direct result of an individual's coping strategies or adaptive skills.
- Exclude studies that only mention the presence of a social network without a formal measurement or detailed qualitative description of how that relates to resilience.
