# Supplementary material for: Facilitators of and Barriers to Resilience Among Black Children and Youth in Canada and the United States: Protocol for a Scoping Review
Source: JMIR Res Protoc. 2025 Oct 20;14:e80859. doi: 10.2196/80859 (PMC12583943; doi:10.2196/80859)
Supplement: Multimedia Appendix 4 [file resprot_v14i1e80859_app4.docx]

| **Author(s) and citation** | **Study Location (Canada or United States)** | **Study Purpose/Objective** | **Population (Children [0-9 years], Adolescents [10-17 years], Youth [18-25 years], no age reported)** | **Research Approach (Qualitative, Quantitative, Mixed Methods)** | **Design/Methodology** | **Sample Size (N)** | **Type of adversity (e.g., anti-Black racism, bullying, racial profiling, etc.)** | **Resilience Dimension (e.g., Process, Capacities, Outcomes)** | **Key Findings** | **Resilience Facilitator(s)** | **Resilience hindrance(s)** | **Implications for future research/Relevant Notes** | **Study Limitation(s)** |
| --- | --- | --- | --- | --- | --- | --- | --- | --- | --- | --- | --- | --- | --- |
|  |  |  |  |  |  |  |  |  |  |  |  |  |  |
|  |  |  |  |  |  |  |  |  |  |  |  |  |  |
|  |  |  |  |  |  |  |  |  |  |  |  |  |  |
|  |  |  |  |  |  |  |  |  |  |  |  |  |  |
|  |  |  |  |  |  |  |  |  |  |  |  |  |  |
|  |  |  |  |  |  |  |  |  |  |  |  |  |  |
|  |  |  |  |  |  |  |  |  |  |  |  |  |  |
|  |  |  |  |  |  |  |  |  |  |  |  |  |  |
|  |  |  |  |  |  |  |  |  |  |  |  |  |  |
|  |  |  |  |  |  |  |  |  |  |  |  |  |  |
|  |  |  |  |  |  |  |  |  |  |  |  |  |  |
|  |  |  |  |  |  |  |  |  |  |  |  |  |  |
|  |  |  |  |  |  |  |  |  |  |  |  |  |  |
|  |  |  |  |  |  |  |  |  |  |  |  |  |  |
|  |  |  |  |  |  |  |  |  |  |  |  |  |  |
|  |  |  |  |  |  |  |  |  |  |  |  |  |  |
|  |  |  |  |  |  |  |  |  |  |  |  |  |  |
|  |  |  |  |  |  |  |  |  |  |  |  |  |  |
|  |  |  |  |  |  |  |  |  |  |  |  |  |  |

**Multimedia Appendix 4: Data Extraction Table**
